# Supplementary figures and images for: High-Throughput, Single-Copy Sequencing Reveals SARS-CoV-2 Spike Variants Coincident with Mounting Humoral Immunity during Acute COVID-19
Source: bioRxiv. 2021 Feb 22:2021.02.21.432184. Preprint. [Version 1] doi: 10.1101/2021.02.21.432184 (PMC7924285; doi:10.1101/2021.02.21.432184)

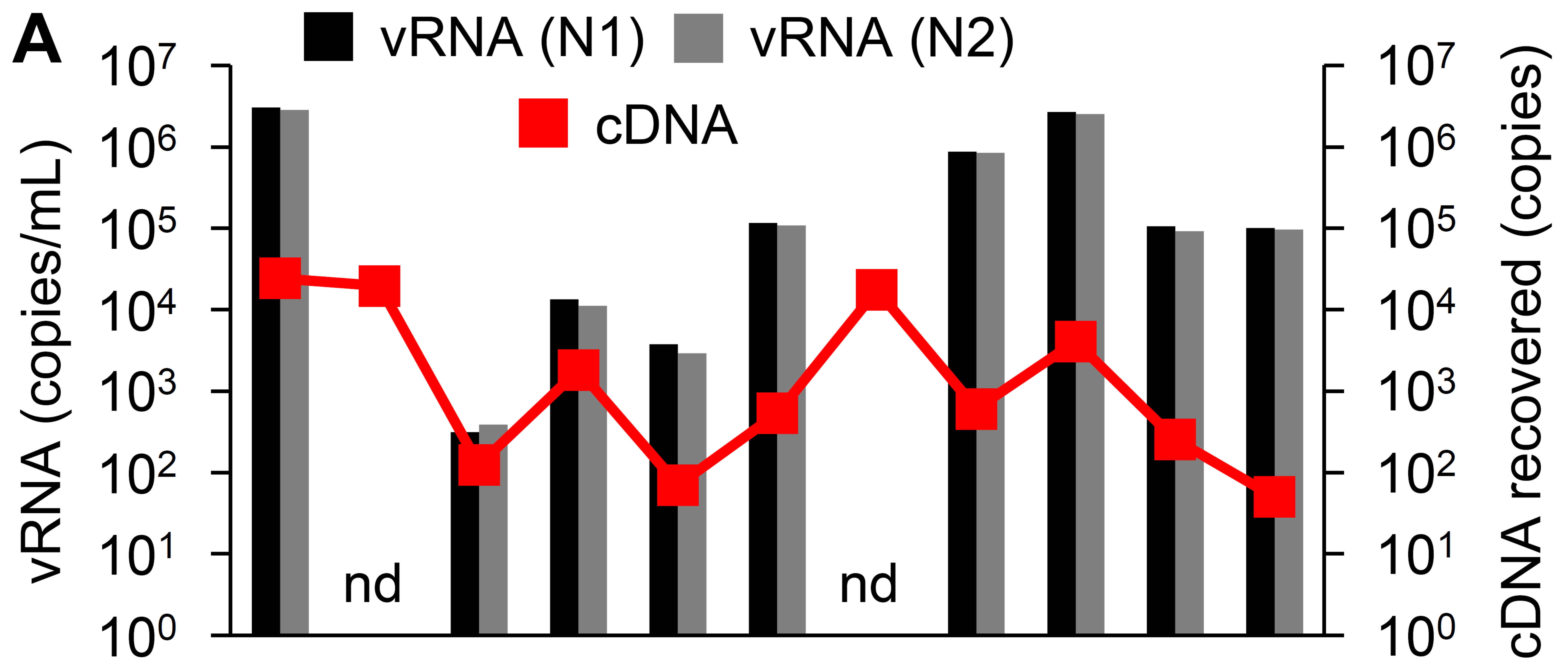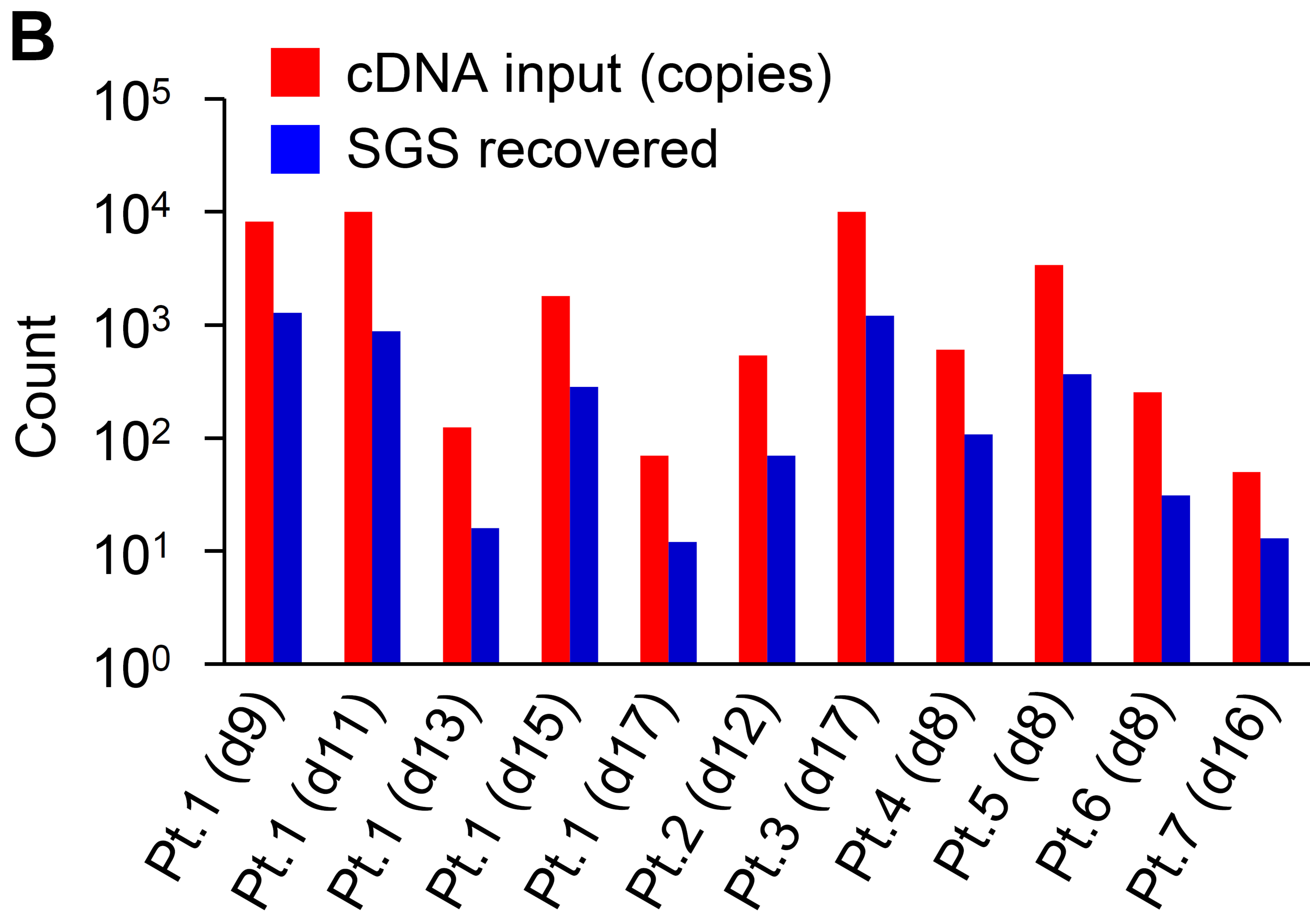

Supplement: Supplement 5 — S3 Fig. Relationships between inputs and yields of steps in the HT-SGS data generation process. (A) Comparison of virus load of original sample with total cDNA synthesis yield. (B) Comparison of cDNA input copies from each sample with final SGS counts. [file media-5.pdf]

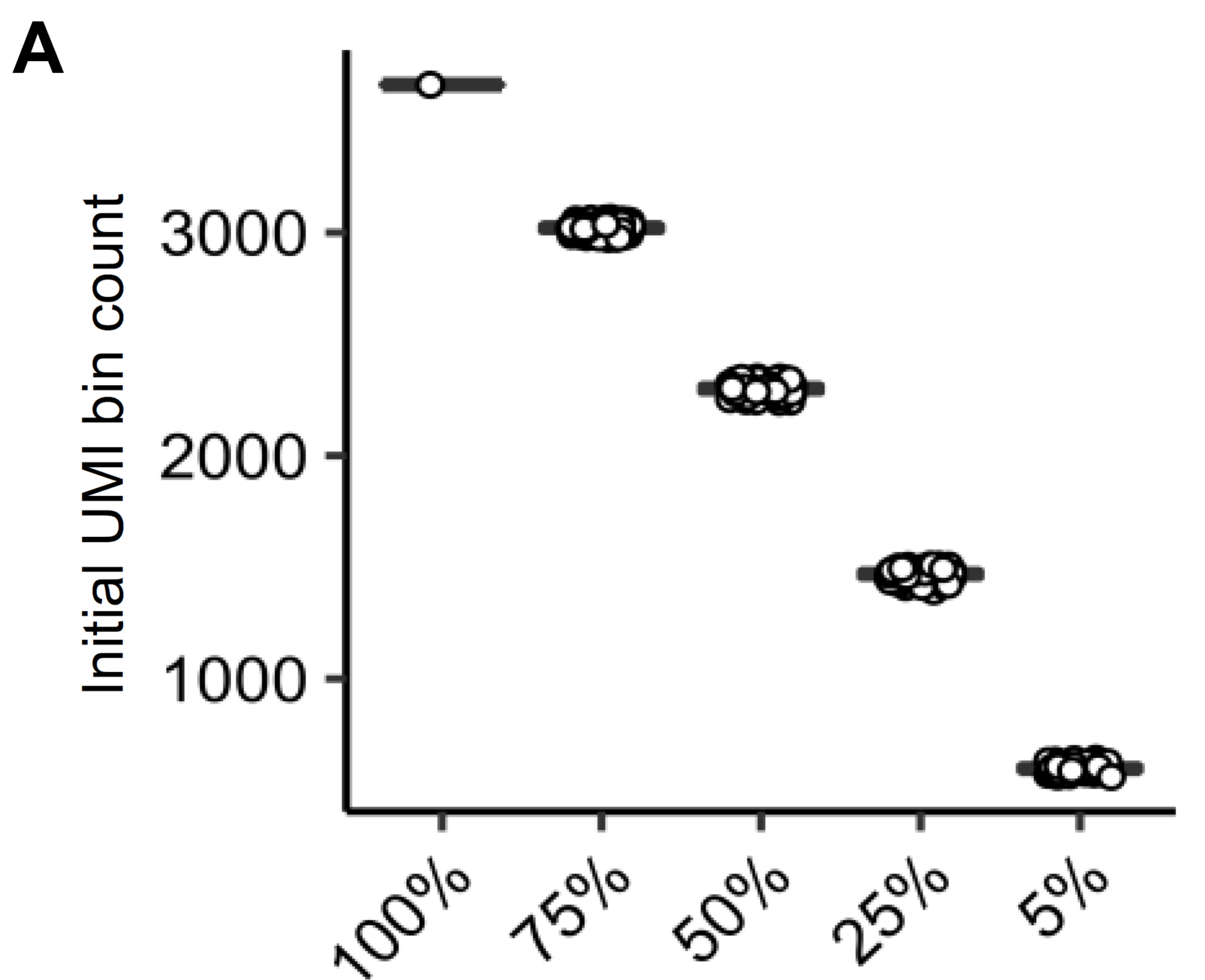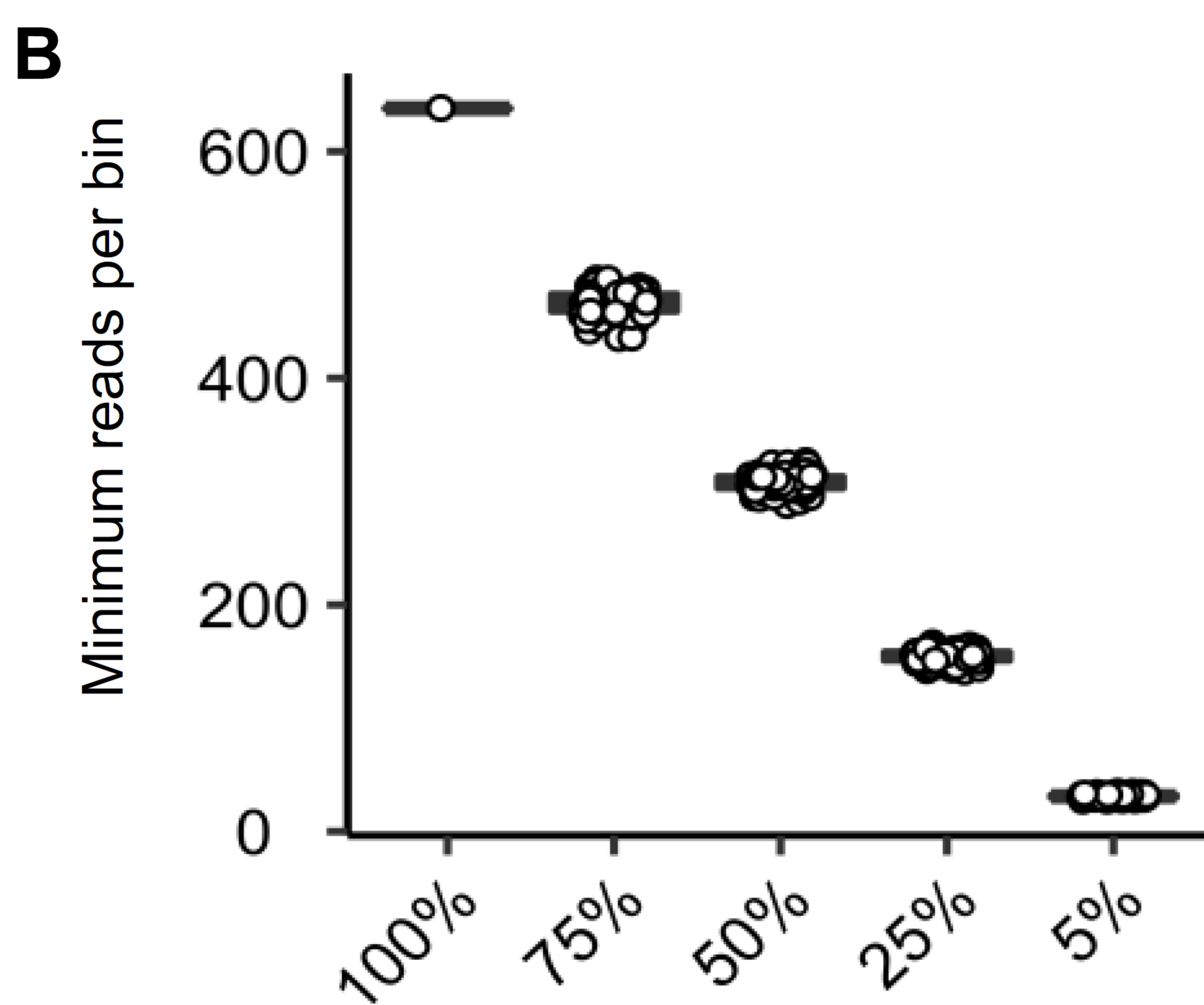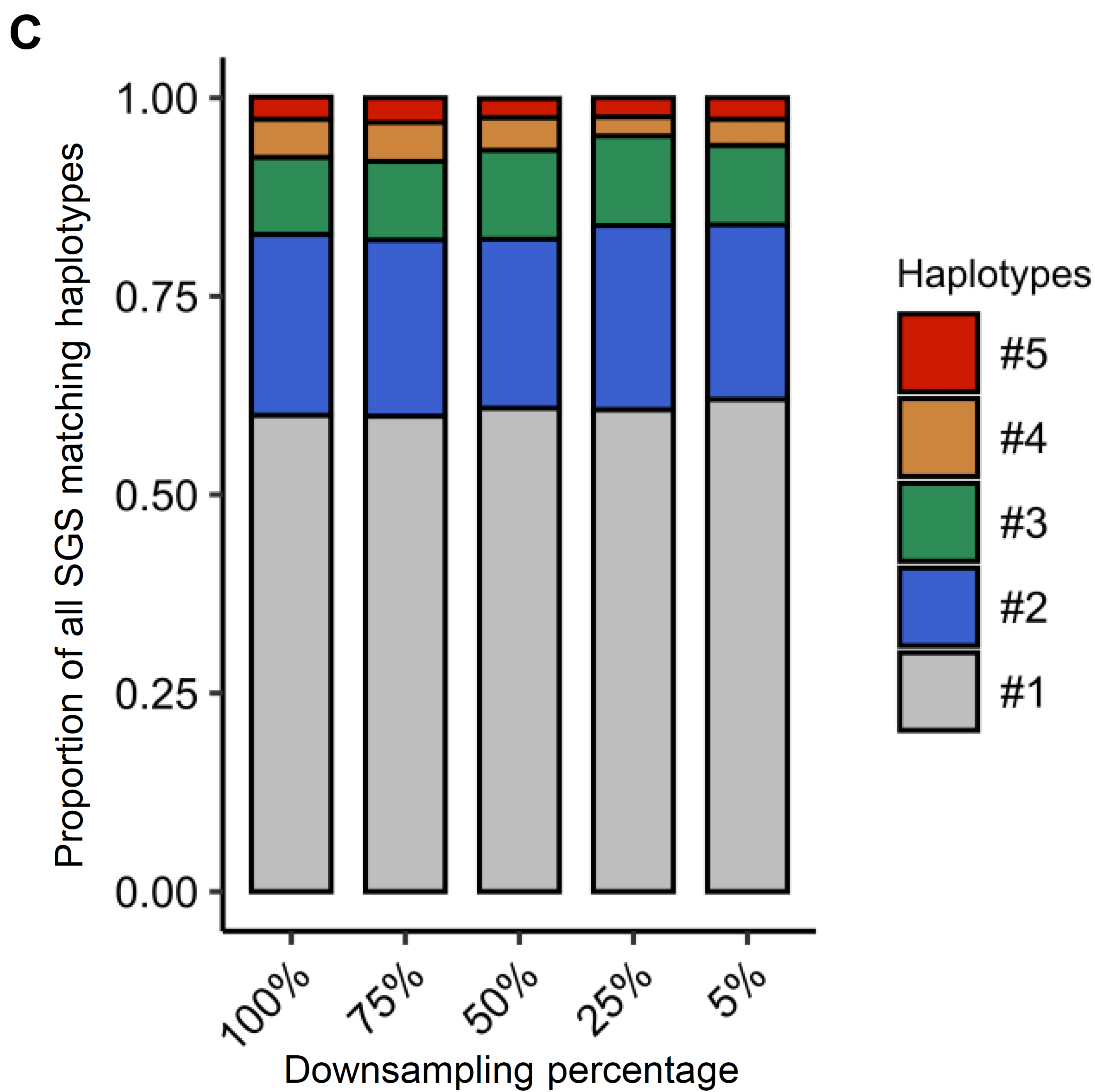

Supplement: Supplement 6 — S4 Fig. Effect of downsampling on haplotype detection. Each subsample was generated by random draws of a fixed percentage from reads without replacement. This process was repeated 100 times for each percentage. (A) The initial numbers of UMI bins (y-axis) are shown for different degrees of downsampling (x-axis). (B) The minimum read counts per UMI bin (y-axis) are shown for different degrees of downsampling (x-axis). (C) Proportion of each haplotype present in the 100% sample and in each subsample. Data analyzed are from sequencing of participant 1, day 15. [file media-6.pdf]
