# Supplementary material for: High-Throughput, Single-Copy Sequencing Reveals SARS-CoV-2 Spike Variants Coincident with Mounting Humoral Immunity during Acute COVID-19
Source: bioRxiv. 2021 Feb 22:2021.02.21.432184. Preprint. [Version 1] doi: 10.1101/2021.02.21.432184 (PMC7924285; doi:10.1101/2021.02.21.432184)
Supplement: Supplement 1 — S1 Fig. Details of HT-SGS process from sample to sequencing. SARS-CoV-2 genomic RNA (gRNA) is reverse-transcribed with a primer that binds in ORF6, downstream of the M gene stop codon, and includes a UMI sequence of 8 random nucleotides flanked by a PCR reverse primer binding site. Reverse-transcription products are amplified by PCR using a forward primer that binds in ORF1, upstream of the spike gene start codon. Amplified products are then subjected to long-read sequencing. [file media-1.pdf]

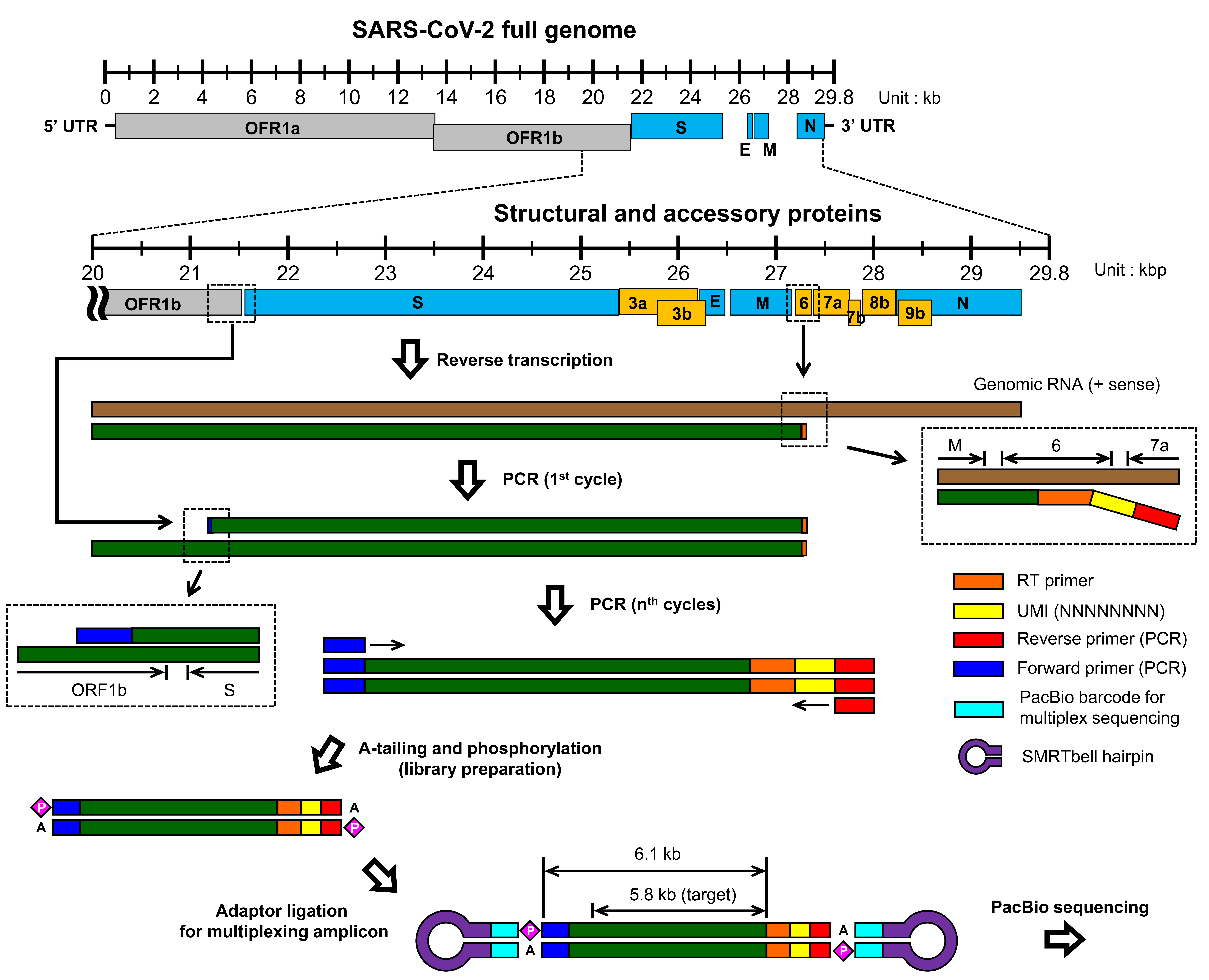

SARS-CoV-2 full genome
0 2 4 6 8 10 12 14 16 18 20 22 24 26 28 29.8
Unit : kb
5' UTR
OFR1a
OFR1b
S
E
M
N
3' UTR
Structural and accessory proteins
20 21 22 23 24 25 26 27 28 29 29.8
Unit : kbp
OFR1b
S
3a
3b
E
M
6
7a
7b
8b
9b
N
Reverse transcription
Genomic RNA (+ sense)
PCR (1st cycle)
M
6
7a
ORF1b
S
PCR (nth cycles)
RT primer
UMI (NNNNNNNN)
Reverse primer (PCR)
Forward primer (PCR)
PacBio barcode for multiplex sequencing
SMRTbell hairpin
A-tailing and phosphorylation
(library preparation)
P
A
Adaptor ligation
for multiplexing amplicon
6.1 kb
5.8 kb (target)
PacBio sequencing
