# Supplementary material for: High-Throughput, Single-Copy Sequencing Reveals SARS-CoV-2 Spike Variants Coincident with Mounting Humoral Immunity during Acute COVID-19
Source: bioRxiv. 2021 Feb 22:2021.02.21.432184. Preprint. [Version 1] doi: 10.1101/2021.02.21.432184 (PMC7924285; doi:10.1101/2021.02.21.432184)
Supplement: Supplement 2 — S1 Table. Clinical characteristics of study participants. [file media-2.pdf]

ID	*Age*	*Sex*	*Race/ Ethn.*	*Sample Day**	*Comorbidity*	*Suppl. Oxygen*	*Pharmacotherapy*	*Outcome*
1	38	F	H	9, 11, 13, 15, 17	LCL, HSCT	None	None	Recovered
2	60	M	AA	12	Chordoma	None	None	Recovered
3	75	M	H	17	Dyslipidemia	MV	Tocilizumab Steroids	Died
4	54	M	W	8	Asthma, CLL	MV	Remdesivir	Recovered
5	67	M	AA	8	Obesity	MV	Steroids Remdesivir	Died
6	22	M	W	8	None	HFNC	None	Recovered
7	62	M	Asian	16	GERD, OSA	MV	None	Recovered

*Sample day denotes day of clinical illness.

H, Hispanic; AA, African-American; W, white; LCL, large cell lymphoma; HSCT, hematopoietic stem cell transplant; CLL, chronic lymphocytic leukemia; GERD, gastro-esophageal reflux disease; OSA, obstructive sleep apnea; MV, mechanical ventilation; HFNC, high-flow nasal cannula; LOS, length of stay.
