# Supplementary material for: High-Throughput, Single-Copy Sequencing Reveals SARS-CoV-2 Spike Variants Coincident with Mounting Humoral Immunity during Acute COVID-19
Source: bioRxiv. 2021 Feb 22:2021.02.21.432184. Preprint. [Version 1] doi: 10.1101/2021.02.21.432184 (PMC7924285; doi:10.1101/2021.02.21.432184)
Supplement: Supplement 3 — S2 Fig. Details of HT-SGS data analysis. (A) Bioinformatic pipeline, depicting sequential workflow steps and tools used. Black boxes show tasks at each step, with the tools used in the grey boxes, and the outputs in the blue bubbles. (B) Initial exclusion of false UMI bins based on read count distribution on a log scale. The dashed line indicates the read count inflection point below which UMI bins in this sample were excluded. (C) Final exclusion of low count UMI bins based on read count distribution on a log scale. The dashed line indicates the read count knee point below which UMI bins in this sample were excluded, following initial false bin removal from the sample and network adjacency. Data are presented for the cultured virus sample presented in Fig 2. [file media-3.pdf]

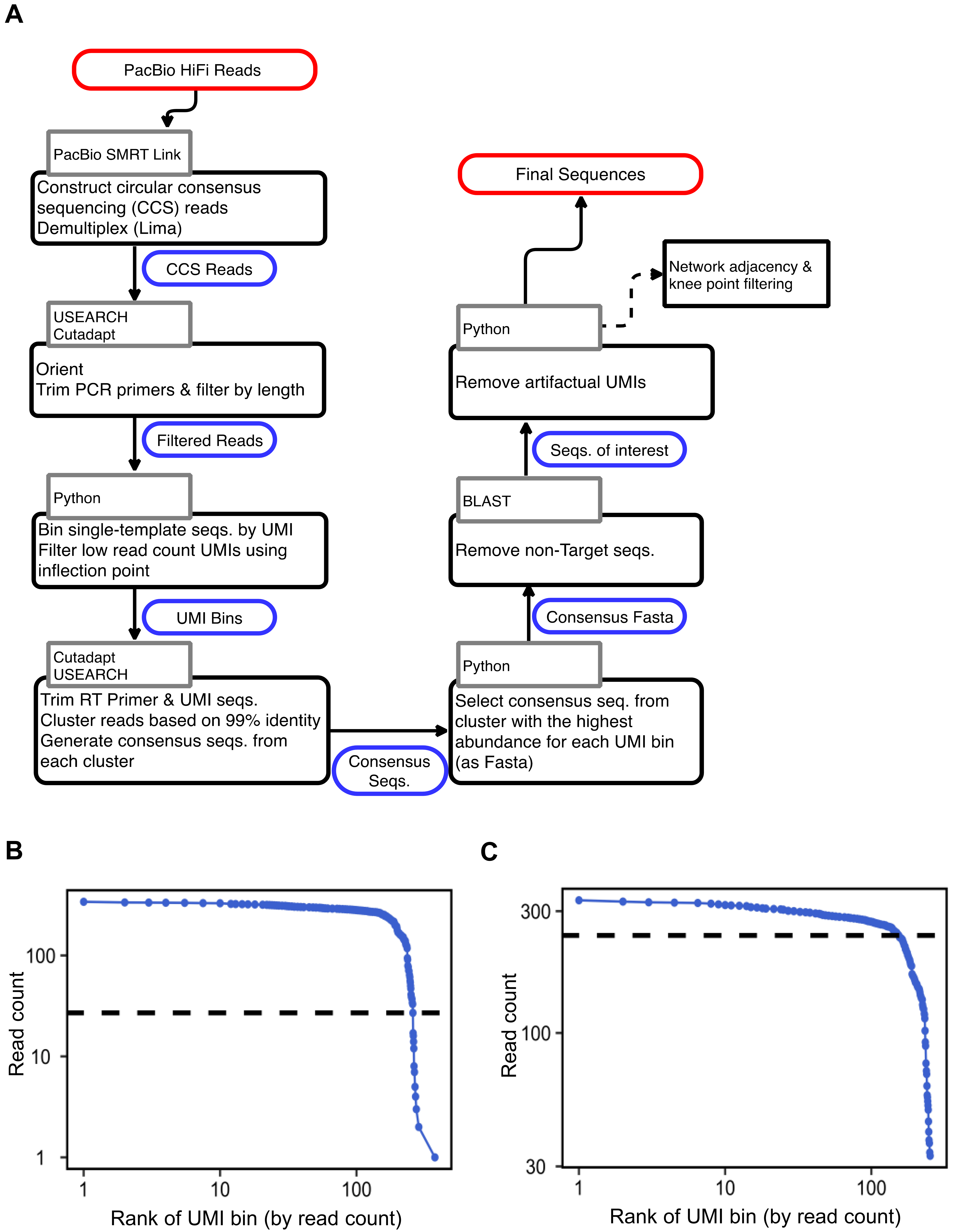
A
PacBio HiFi Reads
PacBio SMRT Link
Construct circular consensus sequencing (CCS) reads
Demultiplex (Lima)
CCS Reads
USEARCH
Cutadapt
Orient
Trim PCR primers & filter by length
Filtered Reads
Python
Bin single-template seqs. by UMI
Filter low read count UMIs using inflection point
UMI Bins
Cutadapt
USEARCH
Trim RT Primer & UMI seqs.
Cluster reads based on 99% identity
Generate consensus seqs. from each cluster
Consensus Seqs.
Python
Select consensus seq. from cluster with the highest abundance for each UMI bin (as Fasta)
Consensus Fasta
BLAST
Remove non-Target seqs.
Seqs. of interest
Python
Remove artifactual UMIs
Network adjacency & knee point filtering
Final Sequences
B
Read count
100
10
1
Rank of UMI bin (by read count)
C
Read count
300
100
30
Rank of UMI bin (by read count)
