# Supplementary material for: High-Throughput, Single-Copy Sequencing Reveals SARS-CoV-2 Spike Variants Coincident with Mounting Humoral Immunity during Acute COVID-19
Source: bioRxiv. 2021 Feb 22:2021.02.21.432184. Preprint. [Version 1] doi: 10.1101/2021.02.21.432184 (PMC7924285; doi:10.1101/2021.02.21.432184)
Supplement: Supplement 4 — S2 Table. Primer sequences used in HT-SGS procedures for this study. [file media-4.pdf]

Primer set	Primer name	MN908947 coordinates (GenBank)	Sequence (5’ → 3’)
Reverse transcription	SEM-RT	27357-27379	CCGCTCCGTCCGACGACTCACTATA NNNNNNNNTCTCCATTGGTTGCTCT TCATCT
FCA forward	FCA_F	22195-22216	TTTAGTGCGTGATCTCCCTCAG
FCA reverse	FCA_R	22588-22608	GATGCAAATCTGGTGGCGTTA
PCR forward-1	PCR_F1	21217-21240	TTCGCATGGTGGACAGCCTTTGTT
PCR forward-2	PCR_F2	21288-21311	TTATCTTGGCAAACCACGCGAACA
PCR reverse	PCR_R	Universal primer	CCGCTCCGTCCGACGACTCACTATA
